# Endolysin LysSte134_1 Reduces Staphylococcus Bacterial Load in Infected Wounds In Vivo

**DOI:** 10.3390/ijms27187989

**Published:** 2026-09-08

**Authors:** Natalia N. Golosova, Yana A. Khlusevich, Bogdana I. Kravchuk, Yuliya N. Kozlova, Timir M. Yakovlev, Svetlana A. Grishkova, Andrey L. Matveev

**Affiliations:** Institute of Chemical Biology and Fundamental Medicine, Siberian Branch of Russian Academy of Sciences, 630090 Novosibirsk, Russiakhlusevichjana@mail.ru (Y.A.K.); semali328@gmail.com (B.I.K.); ulona79@mail.ru (Y.N.K.); t.yakovlev@g.nsu.ru (T.M.Y.); jelikk5@gmail.com (S.A.G.)

**Keywords:** endolysin, *Staphylococcus epidermidis*, *Staphylococcus aureus*, antimicrobial agent, wound infection, wound, LysSte134_1

## Abstract

*Staphylococcus aureus* is an ESKAPE pathogen, defined by its ability to evade antibacterial treatment, and together with *S. epidermidis* is a leading Gram-positive cause of wound infection. The emergence of methicillin- and vancomycin-resistant strains further limits conventional antibiotic treatment. The staphylococcal endolysin LysSte134_1 was produced recombinantly, its proper folding confirmed by zymography, and its lytic activity demonstrated against planktonic cultures of both *S. aureus* and *S. epidermidis*. The combination of LysSte134_1 with 10% 1,3-propanediol and Zn^2+^ increased CFU reduction in both species relative to LysSte134_1 with Zn^2+^ alone. In a murine wound model, topical application of LysSte134_1 significantly reduced the bacterial burden in wound tissue, achieving a 2–3-log reduction in MRSA and below the detection limit for *S. epidermidis*. Treatment was accompanied by normalization of peripheral leukocyte counts and a shift in the serum cytokine profile from a sustained pro-inflammatory state with elevated IL-6, IL-17 and IL-10 toward levels comparable to non-infected controls. These results suggest the potential utility of LysSte134_1 as a component of topical antibacterial formulations and support further investigation in models of staphylococcal wound infection.

## 1. Introduction

*Staphylococcus aureus* and *Staphylococcus epidermidis* are the two staphylococcal species most often recovered from infected wounds [1]. *S. aureus* belongs to the ESKAPE group of pathogens, named for their ability to escape antibacterial action through resistance mechanisms and biofilm-mediated tolerance, and methicillin- (MRSA) and vancomycin-resistant (VRSA) strains are listed by the WHO as high-priority targets for new antimicrobials [2,3]. *S. epidermidis* is not formally part of the ESKAPEE panel, but multidrug-resistant nosocomial isolates have become a clinical problem comparable to *S. aureus* in implant- and healthcare-associated wound infections [4,5,6]. Chronic non-healing wounds, defined by failure to progress through the canonical phases of repair or to achieve at least 50% area reduction within 1–3 months of standard care, place a substantial burden on healthcare systems [7,8,9]. A wound bioburden above 10^5^ colony-forming units per gram of tissue correlates with delayed healing and antimicrobial tolerance, and prolonged hospitalization shifts the wound microbiome toward nosocomial multidrug-resistant lineages, including MRSA [10,11].

*S. aureus* is a coagulase-positive commensal of the skin and nares that becomes invasive after barrier disruption through MSCRAMM-mediated adhesion, biofilm maturation, production of cytotoxins such as α-haemolysin and Panton–Valentine leukocidin, and immune evasion via protein A and coagulase [1]. Expression of these factors is governed by the *agr* quorum-sensing system, while mobile genetic elements drive the emergence of MRSA and vancomycin-resistant lineages [12,13]. In chronic wounds, *S. aureus* sustains the expression of IL-1β, IL-6, TNF-α and CXCL-1, contributing to persistent inflammation and delayed re-epithelialization [10,14,15]. *S. epidermidis*, the principal coagulase-negative staphylococcus of wound origin, follows an opportunistic pattern linked to indwelling devices and healthcare-associated procedures and accounts for 33–37% of monomicrobial orthopedic implant infections [16,17,18]. Its main virulence determinant is biofilm formation, mediated by autolysins and MSCRAMMs in primary attachment and by the *icaADBC*-encoded polysaccharide intercellular adhesin (PIA/PNAG) together with PIA-independent proteins in matrix accumulation [19,20].

The current standard of care for staphylococcal wound infections relies on systemic and topical antibiotics. Its efficacy is increasingly compromised both by acquired resistance, particularly in MRSA and multidrug-resistant *S. epidermidis*, and by biofilm formation: staphylococcal biofilms tolerate antibiotic concentrations up to three orders of magnitude higher than the corresponding planktonic cultures and resist immune clearance [21]. Antibiotic regimens against biofilm-associated wound infections therefore often produce only transient suppression of the bacterial load without eradication of the pathogen [22,23,24].

These limitations motivate the search for antimicrobials with mechanisms different from those of conventional antibiotics. Phage-encoded endolysins are peptidoglycan hydrolases that lyse Gram-positive bacteria when applied externally to the cell wall, combining a narrow species-level spectrum with rapid bactericidal action against planktonic and sessile cells, a low propensity for resistance selection and documented efficacy in preclinical models of cutaneous infection [25,26]. The wound surface is nevertheless an unfavorable environment for protein-based antimicrobials: host and bacterial proteases, variations in pH and ionic strength, exudate dilution and desiccation reduce the activity and residence time of an applied enzyme, and for metal-dependent endolysins the catalytic cation must remain available at the application site [27]. Topical composition with biocompatible polyols such as 1,3-propanediol has been proposed to address these constraints: 1,3-propanediol acts as a humectant, contributes to protein stabilization and has its own antimicrobial activity at moderate concentrations [28,29]. In vivo characterization of formulated endolysin preparations in murine wound infection models is, however, limited.

The endolysin of staphylococcal phage vB_SepP_134 (LysSte134_1) was characterized in our previous work [30]. LysSte134_1 is a Zn^2+^-dependent N-acetylmuramoyl-L-alanine amidase capable of disrupting biofilms formed by *S. aureus* and *S. epidermidis* in vitro [30]. The behavior of LysSte134_1 in a topical composition and its efficacy in vivo have not been examined. In this work, zymography demonstrated lytic activity of LysSte134_1 against peptidoglycans of two additional staphylococcal species, with partial activity against two further species. LysSte134_1 was produced recombinantly, and a topical composition was prepared containing the endolysin, 1,3-propanediol as a humectant and Zn^2+^ at the identified optimal concentration. The composition was applied to infected wounds in a murine model of methicillin-resistant *S. aureus* and *S. epidermidis* infection, and bacterial load in wound tissue, complete blood count and serum interleukin levels were measured to assess local clearance and the systemic response. These findings support further investigation of LysSte134_1 as a component of topical formulations for the treatment of wound infections caused by methicillin-resistant *S. aureus* and *S. epidermidis*.

## 2. Results

### 2.1. Enzymatic Activity of LysSte134_1 by Zymography Assay Against S. simulans, S. auricularis, S. caprae, S. capitis

**Figure 1:**
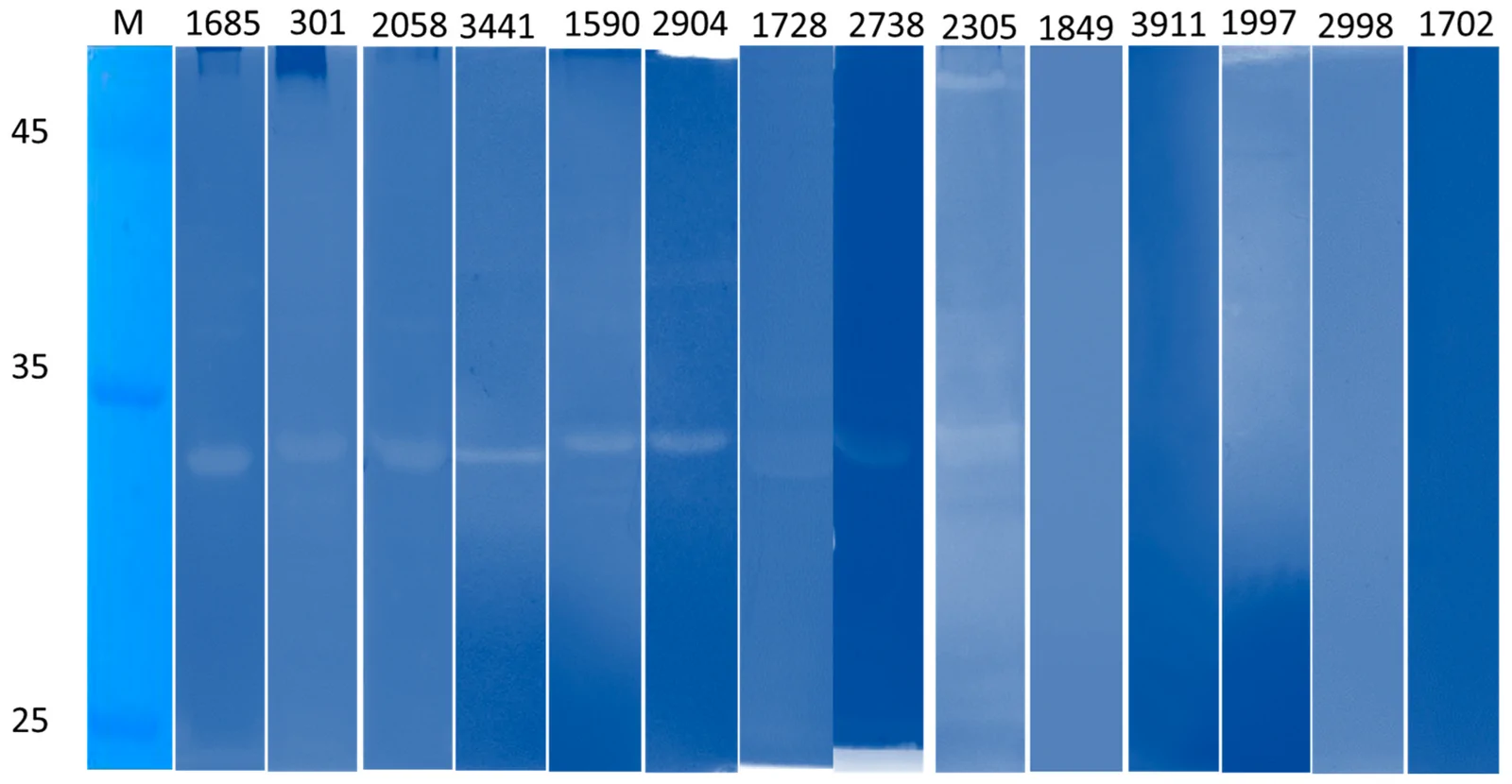
Zymographic analysis of recombinant LysSte134_1. PAGE (12.5%) containing 0.5 mg of peptidoglycans from *S. aureus* CEMTC 1685 (1), *S. epidermidis* CEMTC 301 (2), *S. epidermidis* CEMTC 2058 (3), *S. epidermidis* CEMTC 3441 (4), *S. capitis* CEMTC 1590 (5) and *S. capitis* 2904 (6), *S. simulans* CEMTC 1728 (7), *S. auricularis* CEMTC 2738 (8), *S. caprae* CEMTC 2305, (9) *S. caprae* CEMTC 1849 (10), *S. warneri* CEMTC 3911 (11), *S. warneri* CEMTC 1997 (12), *S. felis* CEMTC 2998 (13) or *S. hominis* CEMTC 1702 (14), stained with methylene blue. M—protein ladder Pierce™ Unstained Protein MW Marker #26610 (Thermofischer, Waltham, MA, USA).

The lytic activity of LysSte134_1 was evaluated by zymography using cell-wall peptidoglycans from various clinical *Staphylococcus* isolates. Peptidoglycans were purified from *S. epidermidis* CEMTC 301, 2058 and 3441, *S. aureus* CEMTC 1685 (VRSA), and *S. capitis* CEMTC 1590 and 2904. LysSte134_1 efficiently hydrolyzed peptidoglycan from these *S. epidermidis*, *S. aureus* and *S. capitis* strains and showed weak lytic activity against *S. simulans* CEMTC 1728, *S. auricularis* CEMTC 2738 (clindamycin-resistant) and *S. caprae* CEMTC 2305. No lytic activity was detected against *S. caprae* CEMTC 1849, *S. warneri* CEMTC 3911 and CEMTC 1997, *S. felis* CEMTC 2998 or *S. hominis* CEMTC 1702 (Figure 1).

### 2.2. Analysis of Antibacterial Activity of LysSte134_1 Against Staphylococcal Planktonic Cells In Vitro

**Figure 2:**
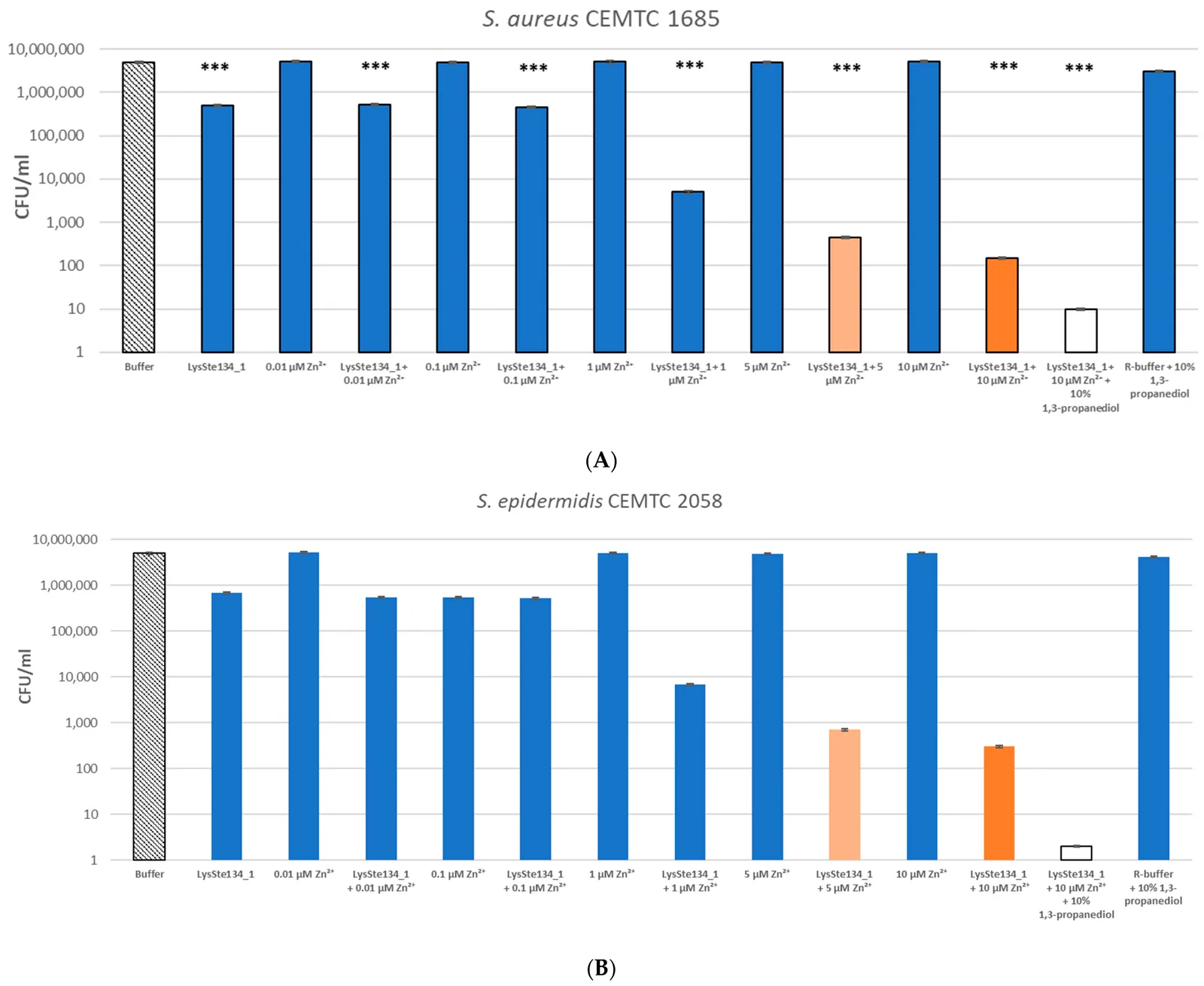
Antibacterial activity of LysSte134_1 against *S. aureus* CEMTC 1685 (**A**) and *S. epidermidis* CEMTC 2058 (**B**). LysSte134_1 (20 µg/mL) in R-buffer was added to staphylococcal cells under the following conditions: R-buffer alone; R-buffer supplemented with 10% (*v*/*v*) 1,3-propanediol; R-buffer supplemented with 0.01, 0.1, 1, 5, or 10 µM ZnCl_2_; or R-buffer containing 10 µM ZnCl_2_ combined with 10% (*v*/*v*) 1,3-propanediol. R-buffer preparations containing the corresponding concentrations of zinc ions and/or 1,3-propanediol were used as negative controls. Statistical analysis was performed using one-way ANOVA followed by Tukey’s multiple-comparisons test. All LysSte134_1-treated groups differed significantly from the positive control (cells not treated with LysSte134_1) and from their respective negative controls (*** *p* < 0.0001). The group treated with LysSte134_1 in the presence of 10 µM Zn^2+^ and 10% 1,3-propanediol differed significantly from both the corresponding R-buffer control containing 10 µM Zn^2+^ and 10% 1,3-propanediol and the LysSte134_1-treated group containing 10 µM Zn^2+^ alone. No significant differences were observed among the conditions with LysSte134_1 containing 1, 5, and 10 µM Zn^2+^ (ns, *p* > 0.99).

For the application of the endolysin in the treatment of staphylococcal wound infections, 1,3-propanediol was used as a moisturizing agent. The influence of 1,3-propanediol on LysSte134_1 enzymatic activity was evaluated against *S. aureus* CEMTC 1685 and *S. epidermidis* CEMTC 2058 using a serial dilution assay. The optimal Zn^2+^ concentration required for LysSte134_1 function was also determined. Treatment of *S. aureus* CEMTC 1685 or *S. epidermidis* CEMTC 2058 cells with LysSte134_1 (20 µg/mL) resulted in a 40-fold reduction in CFU (Figure 2). Subsequently, the impact of varying Zn^2+^ levels on LysSte134_1 efficacy was assessed. In the presence of ZnCl_2_ at 1 µM, 5 µM, or 10 µM, the lytic effect of LysSte134_1 on both strains increased to 100-, 1000-, and 5000-fold, respectively (Figure 2). Lower ZnCl_2_ concentrations (0.01 µM and 0.1 µM) enhanced LysSte134_1 activity by 5- and 25-fold. The addition of 10% 1,3-propanediol led to a further increase in the antibacterial effect of LysSte134_1 by at least one order of magnitude, and for *S. epidermidis* CEMTC 2058 reduced the pathogen to below the limit of detection of viable cells.

### 2.3. Hemolytic Activity of LysSte134_1

**Figure 3:**
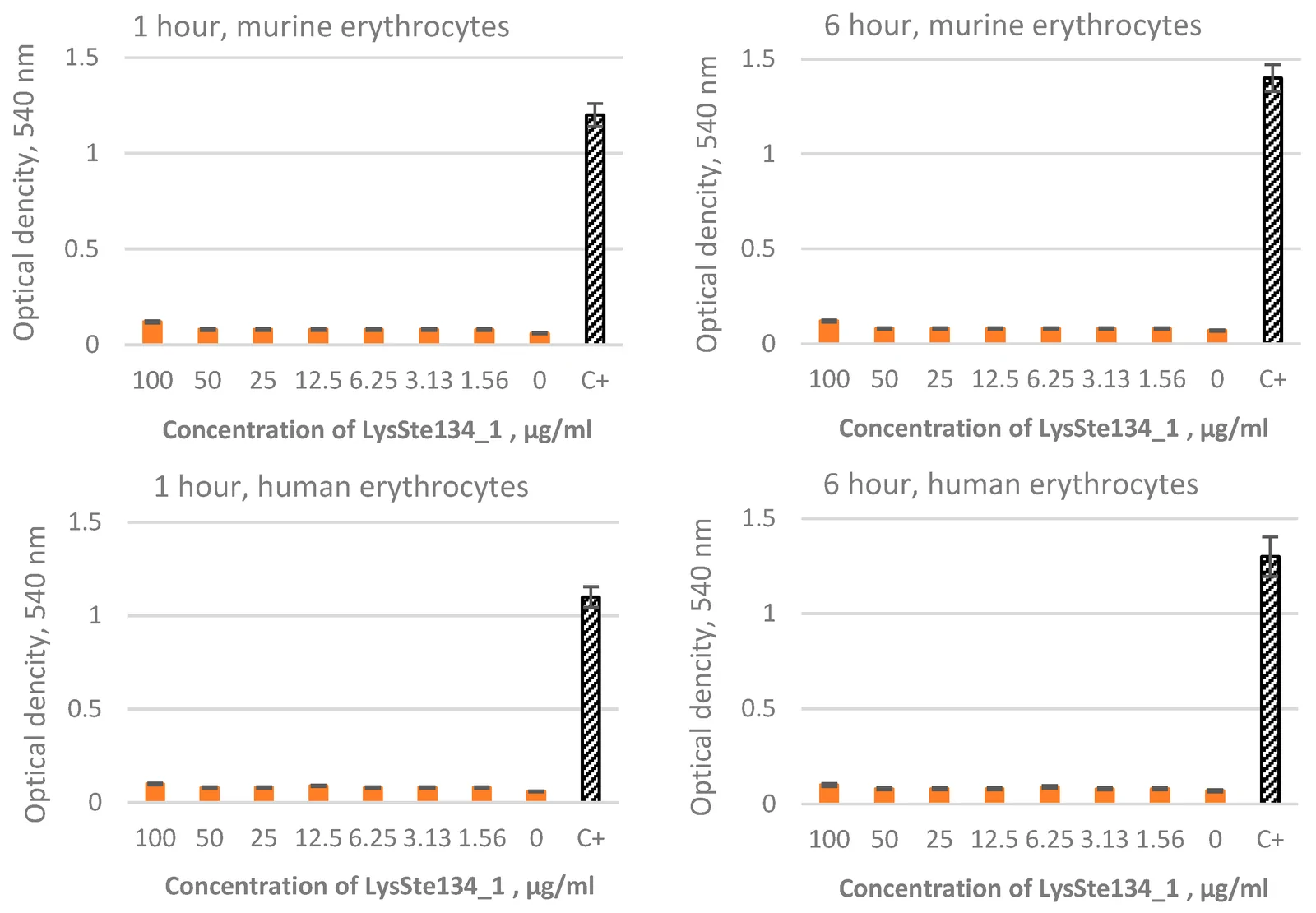
Hemolytic activity of recombinant endolysin LysSte134_1. Human and murine erythrocytes were incubated with LysSte134_1 in seven two-fold serial dilutions starting at 100 µg/mL for 1 h and 6 h at 37 °C. Erythrocytes in buffer without protein served as a negative control, and erythrocytes treated with 1% Tween-20 served as a positive control. Statistical analysis was performed using one-way ANOVA; all tested groups differed significantly from the positive control group, *p* < 0.0001.

LysSte134_1 was tested for hemolytic activity on human and mouse erythrocytes over a wide concentration range (seven concentrations starting from 100 µg/mL) and at two incubation times (1 h and 6 h). Across all tested concentrations and time points, LysSte134_1 did not induce detectable hemolysis of either human or murine erythrocytes, with values remaining at the level of the negative control and far below those observed with the positive control. These data indicate that LysSte134_1 lacks direct hemolytic activity under the experimental conditions used (Figure 3).

### 2.4. In Vivo Antibacterial Activity of LysSte134_1 in a Murine Wound Infection Model

The antibacterial activity of the recombinant endolysin LysSte134_1 was assessed in a murine model of purulent staphylococcal wound infection. Mice were assigned to six groups (*n* = 6 per group): intact uninfected animals without a wound (Intact), animals with a non-infected wound (Non-infected wound), infected untreated animals (Untreated), infected animals treated with vehicle (0.9% NaCl; NaCl), infected animals treated with the endolysin-free composition (Composition), and infected animals treated with the LysSte134_1-containing composition (LysSte134_1, 1 mg/mL). Wounds were infected with either MRSA CEMTC 675 or *S. epidermidis* CEMTC 2058, and therapy was initiated after formation of an open purulent lesion.

In *S. aureus-*infected wounds, treatment with the LysSte134_1-containing composition resulted in a substantial reduction in bacterial burden. The *S. aureus* titer in purulent wounds decreased by 500 ± 32-fold compared with untreated animals and by 2000 ± 84-fold compared with mice receiving the endolysin-free composition (*p* < 0.0001 for both comparisons; two-way ANOVA followed by Tukey’s HSD post hoc test). In contrast, high bacterial loads persisted in both control groups, indicating that the vehicle alone did not provide a meaningful antibacterial effect.

**Figure 4:**
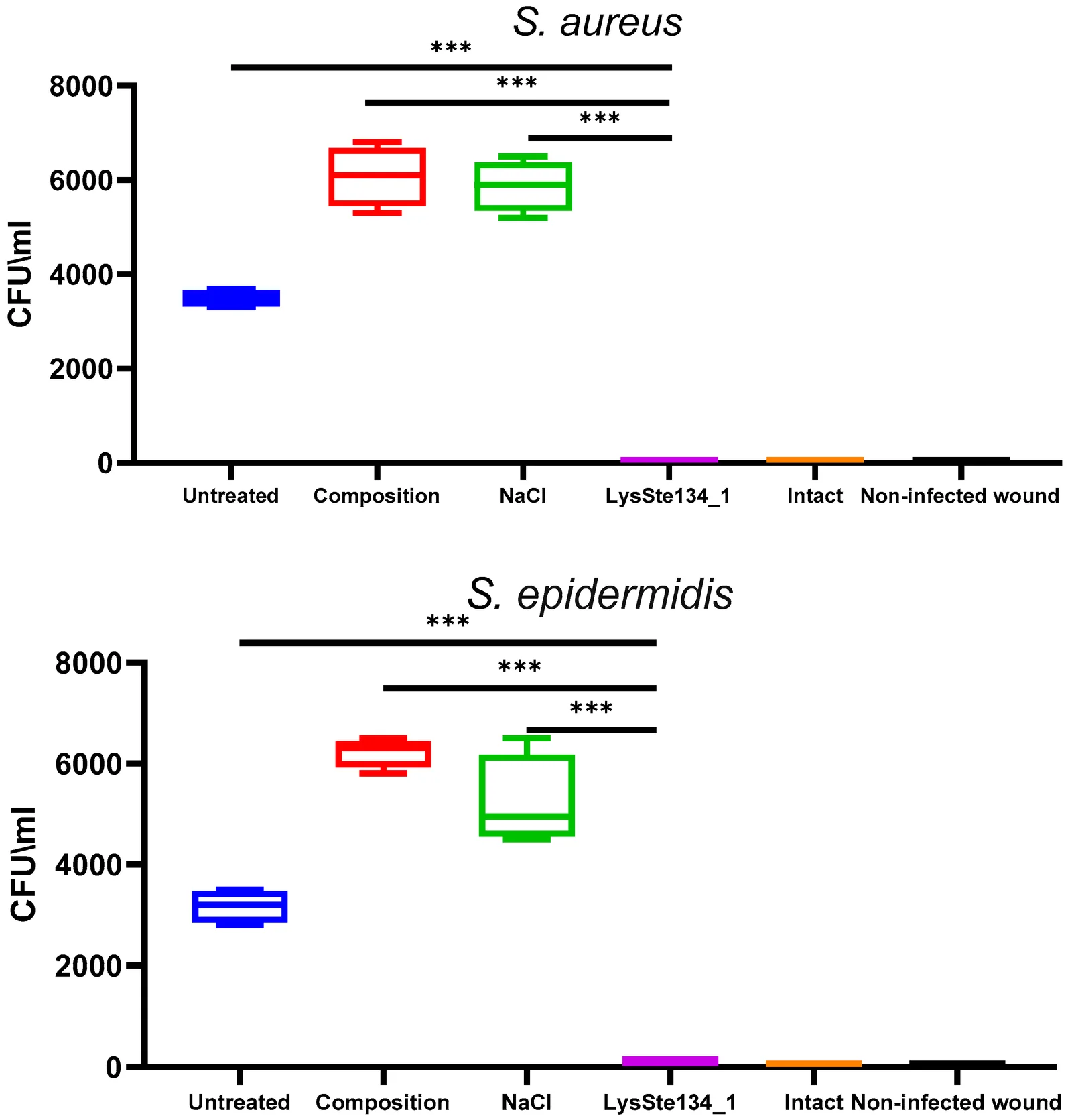
Bacterial burden in wound tissues at day 14 post-infection. Bacterial counts (CFU/mL) were determined in wounds infected with *S. aureus* CEMTC 675 or *S. epidermidis* CEMTC 2058. Experimental groups included untreated infected mice (Untreated), mice treated with the endolysin-free formulation (Composition), mice treated with 0.9% NaCl (NaCl), mice treated with the LysSte134_1-containing formulation (LysSte134_1), intact uninfected animals without wounds (Intact), and animals with non-infected wounds (Non-infected wound). Data are presented as mean ± SD. Statistical analysis was performed using two-way ANOVA followed by Tukey’s multiple-comparisons test. The LysSte134_1-treated group showed significantly lower bacterial counts than the Untreated, Composition, and NaCl groups for both bacterial species (*** *p* < 0.0001). No significant difference was observed between the LysSte134_1 and Intact groups (*S. aureus*, *p* > 0.9999; *S. epidermidis*, *p* = 0.9987).

In wounds infected with *S. epidermidis*, application of LysSte134_1 led to below the limit of detection of the pathogen from the wound site. *S. epidermidis* titers in the untreated group reached (3 ± 0.54) × 10^4^ CFU/mL, and in animals treated with the endolysin-free composition they were (6 ± 0.73) × 10^4^ CFU/mL, whereas no colonies were detected in the LysSte134_1-treated group. No bacterial growth was observed in the intact group. Across all groups, CFU counts obtained on LB agar and mannitol salt agar were comparable, confirming the consistency of the microbiological readout (Figure 4).

**Figure 5:**
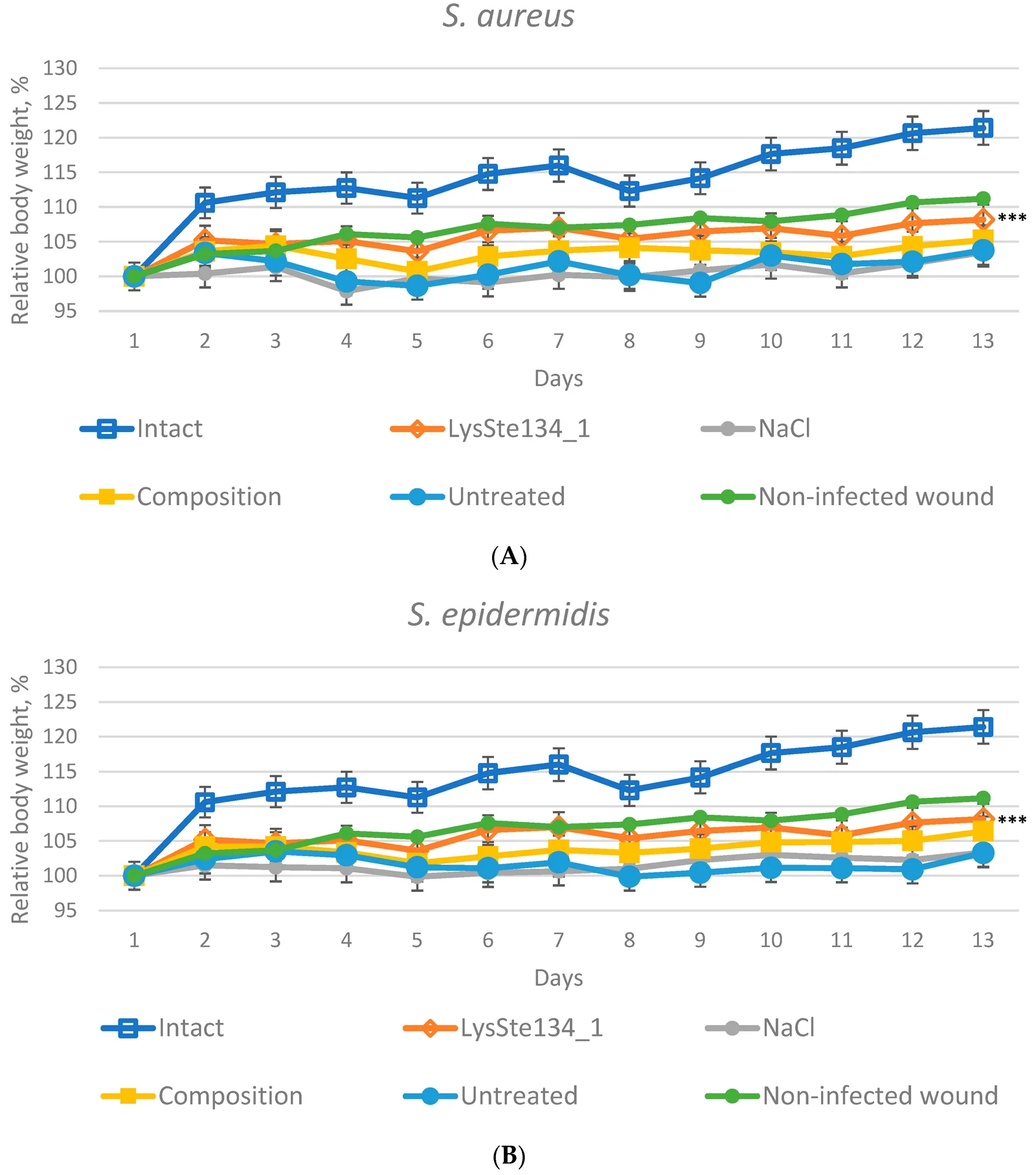
Relative body weight changes in murine wound-infection models caused by *S. aureus* (**A**) and *S. epidermidis* (**B**). Body weight is expressed as a percentage of the baseline value recorded on day 1 (100%) and is presented as mean ± SD for days 1–13. Six groups were included: Intact, Non-infected wound, NaCl, Untreated, Composition, and LysSte134_1 (*n* = 6 animals per group). Data were analyzed by two-way repeated-measures ANOVA, with Group as the between-subjects factor and Time as the within-subjects factor; the Greenhouse–Geisser correction was applied when sphericity was violated (Mauchly’s test, *p* < 0.001). For both infection models, significant effects of Time, Group, and the Time × Group interaction were observed (*p* < 0.001 for all). In the *S. aureus* model, body weight increased significantly faster in the LysSte134_1 group than in the NaCl and Untreated groups (both *p* < 0.001). Animals receiving the endolysin-free composition showed a slower weight gain than the Non-infected wound group (*p* = 0.003) and the LysSte134_1 group (*p* = 0.023). In the *S. epidermidis* model, the LysSte134_1 group gained weight significantly faster than the NaCl (*p* = 0.034) and Untreated groups (*p* < 0.001), whereas NaCl-treated animals gained weight more slowly than animals with non-infected wounds (*p* = 0.004). *** *p* < 0.001.

Body weight changes reflected the course of infection and the impact of therapy. In *Staphylococcus* spp.-infected animals, body weight started to decline from day 1 post-infection compared with intact controls, with a maximal difference of 6.5% observed on day 2. In the untreated groups, this weight loss persisted for up to 6 days, followed by a gradual recovery (Figure 5). In contrast, in mice receiving the LysSte134_1-containing composition, weight loss ceased within 1 day after the onset of therapy (day 4 post-infection), and body weight then increased steadily until the end of the observation period. Similar trends were observed in both *S. aureus*- and *S. epidermidis*-infected cohorts, indicating that LysSte134_1 treatment not only reduced bacterial load but also reduced the effect of the infection on body weight (Figure 5).

**Figure 6:**
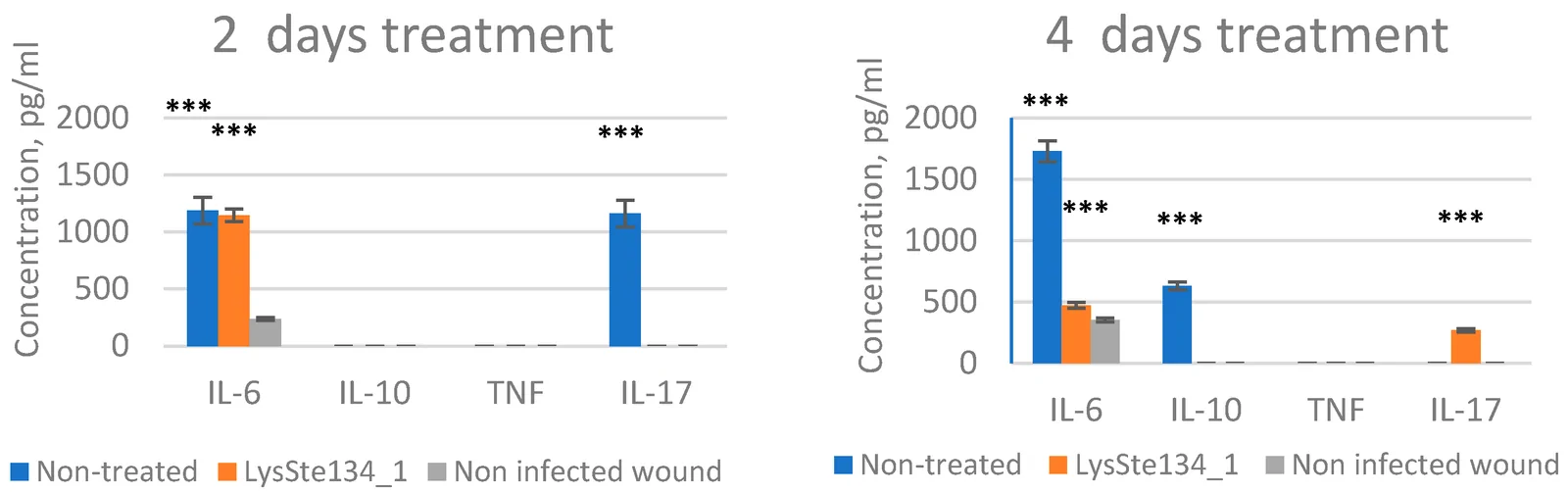
Serum concentrations of IL-6, IL-10, TNF-α, and IL-17 were determined by ELISA and are presented as mean ± SD. IL-6, IL-10, and IL-17 data were analyzed by two-way repeated-measures ANOVA followed by Tukey’s HSD test and paired *t*-tests. Significant effects of Group and Group × Day interaction were observed for IL-6, IL-10, and IL-17 (*p* < 0.001). On day 2, IL-6 was elevated in the Untreated and LysSte134_1 groups compared with the Non-infected wound group (both *p* < 0.001). By day 4, IL-6 increased further in untreated animals (*p* = 0.001) but decreased in the LysSte134_1 group (*p* < 0.001); IL-6 levels differed between these groups on day 4 (*p* < 0.001). IL-10 was undetectable on day 2 in all groups and was detected only in untreated animals on day 4 (631.2 ± 20.0 pg/mL; *p* < 0.001 vs. LysSte134_1 and Non-infected wound). IL-17 was elevated only in untreated animals on day 2 (*p* < 0.001), declined to the detection limit by day 4, and became detectable in the LysSte134_1 group on day 4 (*p* < 0.001). Asterisks indicate significant differences from the Non-infected wound group at the corresponding time point (*** *p* < 0.001).

Two and four days after the start of treatment, hematological parameters and cytokine profiles differed between untreated mice and animals receiving LysSte134_1 (Table 1 and Table 2, Figure 6). On day 2, total WBC counts were elevated in the infected untreated animals and in animals with a non-infected wound relative to intact controls, consistent with an early inflammatory response to wounding and infection, whereas LysSte134_1-treated animals showed WBC counts close to the intact level. By day 4, WBC counts in the LysSte134_1-treated group remained close to those of intact animals (15.07 ± 3.78 versus 17.17 ± 10.88 × 10^9^/L), whereas in untreated animals the early leukocytosis was followed by a pronounced fall in total WBC count (6.92 ± 4.54 × 10^9^/L), the lowest value among all groups. This biphasic pattern in untreated animals, an initial rise followed by a decline below the intact reference, is consistent with leukocyte consumption under a persistent, uncontrolled bacterial load, whereas the stable WBC counts in LysSte134_1-treated animals paralleled the clearance of the wound bacterial burden (Table 2). Serum cytokine analysis showed that on day 2 untreated mice had elevated IL-6 and IL-17 levels, consistent with an active systemic pro-inflammatory response, whereas IL-17 was not detectable in animals treated with LysSte134_1 (Figure 6). By day 4, IL-6 concentrations in untreated animals increased further and were accompanied by pronounced IL-10 production, while in LysSte134_1-treated mice IL-6 levels declined to values close to those in non-infected wound controls, IL-10 remained undetectable, and IL-17 concentrations rose to a moderate level, indicating resolution of systemic inflammation in parallel with bacterial elimination. By the final day of observation, wound area was reduced relative to the first day in all wounded groups (Table 3). The largest reduction occurred in the Non-infected wound group. Among the infected groups, wound area tended to be smaller in animals receiving LysSte134_1; this reached significance only versus untreated animals in the *S. epidermidis* model (*p* = 0.030) and remained a non-significant trend in the other comparisons.

## 3. Discussion

The widespread emergence of multidrug-resistant (MDR) bacteria has reduced the efficacy of conventional therapies for wound infections and increased the number of patients with chronic wounds in which bacterial biofilms form within the wound bed. The declining effectiveness of standard treatments has stimulated the search for alternative systemic and topical antimicrobial approaches. One such approach is bacteriophage therapy, which can selectively target MDR pathogens while maintaining a favorable safety profile and stability under diverse conditions. However, the narrow host range of bacteriophages requires individual phage selection, making treatment labor-intensive and time-consuming. Phage-derived enzymes, including endolysins, represent an alternative approach because they directly degrade the bacterial cell wall. Endolysins can lyse both planktonic bacteria and biofilm-associated cells and are increasingly considered a promising class of antimicrobial agents [31,32,33,34,35,36,37,38,39,40,41,42,43,44].

In our previous study, LysSte134_1 was shown to hydrolyse the cell walls of *S. aureus*, *S. epidermidis*, *Staphylococcus haemolyticus*, and *Staphylococcus warneri*, to lyse planktonic *S. aureus* and *S. epidermidis* cells, and to disrupt biofilms formed by clinical isolates of these species. Because wound colonization and infection may also involve staphylococcal species other than *S. aureus*, the present study expanded the panel of coagulase-negative staphylococci commonly found on human skin to evaluate their susceptibility to LysSte134_1. The recombinant endolysin hydrolysed the cell-wall peptidoglycan of *Staphylococcus simulans*, *Staphylococcus auricularis*, *Staphylococcus caprae*, and *Staphylococcus capitis*.

The haemolytic activity of LysSte134_1 was assessed in this study. No haemolysis of human or murine erythrocytes was detected under the tested conditions, indicating that LysSte134_1 did not lyse host erythrocytes at the concentrations and incubation times used. Cytotoxicity towards keratinocytes, fibroblasts, macrophages, and other skin-resident cells was not evaluated. Topical administration of LysSte134_1 in a 1,3-propanediol-based formulation reduced bacterial burden in MRSA-infected wounds and decreased *S. epidermidis* counts to below the limit of detection. Treatment was also accompanied by recovery of body weight in treated animals.

The hematological and cytokine findings suggest that the observed effects of LysSte134_1 may be primarily associated with bacterial-load reduction and accompanying changes in systemic inflammatory markers. In untreated animals, infection was associated with a marked systemic response, including leukocytosis and increased IL-6 and IL-17 levels at early time points after treatment initiation, followed by concurrent elevation of IL-6 and IL-10 at later stages. In contrast, in LysSte134_1-treated mice, WBC counts approached those of non-infected controls by day 4, IL-6 levels decreased, no increase in IL-10 was observed, and IL-17 remained at moderate levels. Together with the reduced bacterial burden in wound tissue and body-weight recovery, these observations are consistent with attenuation of the systemic inflammatory response during treatment of staphylococcal wound infection with recombinant LysSte134_1.

Direct effects on wound-closure kinetics and tissue remodeling were not assessed, and the small group size (*n* = 6 per group) limits the statistical resolution of secondary hematological and cytokine endpoints. Wound area was evaluated as the change in defect size between the first and last days of observation. Wound area tended to be smaller in the LysSte134_1-treated group than in the untreated, NaCl and endolysin-free composition groups; this difference reached significance only versus untreated animals in the *S. epidermidis* model and remained a non-significant trend in the other comparisons. However, the absence of intermediate measurements precluded reconstruction of the full-time course of wound closure and limited interpretation of tissue-regeneration dynamics. These findings should therefore be regarded as preliminary evidence of a possible effect of the LysSte134_1-containing formulation on wound area in this model. Further studies should include additional time points and morphological assessment of reparative processes.

The present findings can be considered in the context of other phage endolysins investigated as topical antibacterial agents against wound infections caused by Gram-positive and Gram-negative pathogens. The endolysin CHAPK-SH3blys, a chimera comprising the CHAP domain of phage K endolysin LysK and the cell-wall-binding SH3b domain of lysostaphin, and the staphylococcal endolysin LysSYL were effective against MRSA-infected wounds under in vitro conditions mimicking the wound environment. The modified lysin GRC-ML07 showed antibacterial activity in immunocompromised mice with *P. aeruginosa*-infected wounds. The *Klebsiella pneumoniae* phage endolysin PlyKp104 reduced bacterial burden in infected mouse wounds. In addition, LysECD7-SMAP, formulated as a hydroxyethylcellulose gel, reduced bacterial loads in diabetes-associated wound infections in db/db mice and in infected burn wounds in rats [45,46,47,48,49].

Against this background, LysSte134_1 differs from most staphylococcal wound-active endolysins in its domain architecture. Endolysins such as XZ.700 and CHAPK-SH3blys use an N-terminal CHAP endopeptidase domain for exogenous lysis, whereas LysSte134_1 contains a Zn^2+^-dependent Amidase_2 (NALAA-2) domain together with an SH3-like cell-wall-binding domain. Other staphylococcal endolysins, including LysK and LysGH15, also contain an Amidase_2 domain but additionally possess a CHAP domain [50,51]. LysSte134_1 also differs in its target spectrum. Chimeric endolysins such as XZ.700 achieved substantial reductions in *S. aureus* counts on reconstructed human epidermis and in murine skin-infection models, but their target range was restricted to this species. In contrast, LysSte134_1 retained activity against *S. epidermidis* and several other coagulase-negative staphylococcal species, which may be relevant to wound infections involving multiple *Staphylococcus* species. Nevertheless, a broader spectrum is not necessarily an advantage: selective agents such as XZ.700 have been developed partly to preserve commensal coagulase-negative staphylococci and maintain the wound microbiome [52]. Thus, the value of genus-level activity may depend on whether the infection is monomicrobial or polymicrobial.

This study has several limitations. First, the in vivo experiments used one murine wound-infection model and clinical isolates *S. aureus* CEMTC 675 (multidrug-resistant MRSA) and *S. epidermidis* CEMTC 2058. Activity against a geographically broader, biofilm-stratified panel of clinical MRSA and multidrug-resistant *S. epidermidis* isolates, including wound- and implant-derived strains from independent collections, was not evaluated. Such studies will be needed to define the potential breadth of clinical applicability.

Second, the infection model was monomicrobial, whereas chronic wounds are often polymicrobial and may include *P. aeruginosa* and members of the order *Enterobacterales*, against which LysSte134_1 is not active. The performance of LysSte134_1 in polymicrobial wound communities remains to be determined. Third, wound outcome was characterized by bacterial burden and macroscopic wound area at two time points; epithelialization, granulation-tissue formation, collagen deposition, angiogenesis, and histopathology were not assessed. Tissue-level characterization will be needed to determine whether the observed antibacterial and inflammatory changes translate into improved tissue repair.

Fourth, the cytokine panel was limited to TNF-α, IL-6, IL-10, and IL-17; wound-healing mediators such as TGF-β1, VEGF, and IL-22 were not measured. Fifth, the in vivo design did not include separate groups receiving Zn^2+^ alone, 1,3-propanediol alone, or their combination without endolysin. The contribution of these components was investigated in vitro (Figure 2), but a full factorial analysis was not performed in vivo. The storage stability and shelf-life of the composition were likewise not characterized and will need to be established for practical application. Sixth, no direct comparison was made with standard topical anti-staphylococcal agents, such as mupirocin or fusidic acid. Comparative efficacy data are needed before drawing translational conclusions.

Seventh, cytotoxicity of LysSte134_1 towards keratinocytes, fibroblasts, macrophages, and endothelial cells was not assessed. The safety assessment was limited to the absence of detectable hemolysis of human and murine erythrocytes under the tested conditions. Comprehensive local and systemic toxicology studies, together with pharmacokinetic characterization, will be required before potential clinical application can be considered. Eighth, hematological and cytokine outcomes were obtained from groups of *n* = 6 animals at two post-treatment time points, which limits the statistical resolution of these secondary endpoints. Dedicated time-course studies with larger cohorts will be required to characterize leukocyte and cytokine responses to topical LysSte134_1 treatment.

Overall, these findings support further investigation of the Zn^2+^-dependent endolysin LysSte134_1 formulated with 1,3-propanediol as a component of a topical approach to staphylococcal wound infections. Its activity against planktonic and biofilm-associated staphylococci, species specificity, absence of detectable hemolysis under the tested conditions, reduction in wound bacterial burden, and accompanying changes in peripheral leukocyte counts and serum cytokine concentrations warrant additional preclinical evaluation. Future studies should include direct comparison with standard topical antibacterial, histopathological assessment of wound repair, evaluation in polymicrobial models, testing against a broader panel of clinical isolates, cytotoxicity assessment in skin-resident cells, and comprehensive local and systemic toxicological and pharmacokinetic characterization.

## 4. Materials and Methods

### 4.1. Bacterial Strains and Growth Conditions

*Staphylococcus* and *Escherichia coli* strains were obtained from the Collection of Extremophile Microorganisms and Type Cultures (CEMTC) of ICBFM SB RAS, which is a clinical isolate collection from hospitals in Novosibirsk. The strains used in the present work include *S. aureus* CEMTC 675 (multidrug-resistant MRSA, clinical isolate) and *S. aureus* CEMTC 1685 (VRSA, clinical isolate). *Staphylococcus* species was determined by sequencing the 16S rRNA gene fragment (~1400 bp). Antibiotic resistance has been previously tested using the disk-diffusion method (Oxoid™, Thermo Fisher Scientific, Waltham, MA, USA) in accordance with the EUCAST (version 8.0) recommendations (Table S1).

The following *E. coli* strains were used for protein expression: *E. coli* M15 (pREP4) (F-, Φ80ΔlacM15, thi, lac-, mtl-, recA+, KmRK12 F- lambda- ilvG- rfb-50 rph-1) [malB+] K-12 (λS)).

Bacterial strains were cultivated in lysogeny broth (LB) media/agar (1.5% *w*/*v*) (10 g/L peptone, 5 g/L yeast extract, 10 g/L NaCl, pH: 7.0, Becton Dickinson, Franklin Lakes, NJ, USA). The planktonic bacteria cells were grown in an orbital shaker incubator with 150 rpm at 37 °C (Infors HT, Bottmingen, Switzerland).

The pQE-60/LysSte134_1 plasmid was previously obtained at the Institute of Chemical Biology and Fundamental Medicine (ICBFM SB RAS, Novosibirsk, Russia), was used in this study.

For the in vivo wound infection model, we used *S. aureus* CEMTC 675. This multidrug-resistant MRSA strain is resistant to cefoxitin (β-lactams), erythromycin (macrolides), clindamycin (lincosamides) and gentamicin (aminoglycosides). Genomic analysis further showed that it carries the *mecA*, *blaZ* and *ermC* resistance genes.

### 4.2. Expression and Purification of Recombinant Lysin LysSte134_1

Recombinant LysSte134_1 was expressed using *E. coli* M15 strain containing the pQE-60/LysSte134_1 plasmid, obtained previously [30]. An overnight culture of *E. coli* M15-pQE-60/LysSte134_1 was grown in 5 mL of LB containing 50 μg/mL ampicillin at 37 °C. For protein production, the overnight culture was diluted 1:100 into 100 mL of fresh LB medium with 50 μg/mL ampicillin and incubated at 37 °C until the optical density at 600 nm (OD_600_) reached 0.6–0.7. Expression was then induced using 100 μM of isopropyl β-D-1-thiogalactopyranoside (IPTG). Induction was carried out at 10 °C for 16 h with shaking at 180 rpm. Cells were harvested by centrifugation (5 min, 6000× *g*), resuspended in 50 mM Tris-HCl, 300 mM NaCl, pH 8.0, and disrupted by sonication using a Sonopuls HD 2070 sonicator (Bandelin, Mecklenburg-Vorpommern, Germany). Lysates were fractionated into soluble (cytoplasmic) and insoluble (inclusion body) fractions by centrifugation (15 min, 14,000× *g*). Expression level and localization of recombinant LysSte134_1 were analyzed by 12.5% SDS-PAGE.

Recombinant LysSte134_1 was purified from the soluble cytoplasmic fraction using Ni-NTA agarose (Qiagen, Venlo, The Netherlands) following the manufacturer’s instructions. Briefly, LysSte134_1 was eluted with buffer A (50 mM NaH_2_PO_4_, 300 mM NaCl, 5 mM Tris-HCl, pH 8.0) containing 100 mM imidazole. The purified protein was concentrated using an Amicon Ultra 4 centrifugal filter unit (Millipore, Burlington, MA, USA) with a 10 kDa molecular weight cutoff. The buffer was exchanged for storage buffer (S buffer: 25 mM Hepes, pH 8.0, 150 mM NaCl) by repeated dialysis. Protein purity was quantified using a GelDoc Go gel documentation system (Bio-Rad, Hercules, CA, USA). Protein concentration was determined using a Qubit Protein Assay Kit (Thermo Fisher Scientific, Waltham, MA, USA) on a Qubit 4 fluorometer (Thermo Fisher Scientific, Waltham, MA, USA).

### 4.3. Zymography with Peptidoglycans from Staphylococcal Cells

Peptidoglycan was isolated from the cell walls of *Staphylococcus* spp. using a previously described protocol [30] with modifications. Briefly, an overnight bacterial culture was diluted 1:500 in 1 L of LB medium and grown at 37 °C with shaking at 150 rpm until the optical density at 600 nm (OD_600_) reached 1.0–1.5. Cells were harvested by centrifugation for 10 min at 6000× *g*. The pellet was resuspended in 10 mL of 4 M LiCl, incubated in a boiling water bath for 20 min, and pelleted again by centrifugation. The resulting precipitate was resuspended in deionized water and disrupted by ultrasonication for 25 min at 35% amplitude using a Sonopuls HD 2070 homogenizer (Bandelin, Berlin, Germany). The homogenate was centrifuged at 12,000× *g* for 12 min. The pellet was then resuspended in 12 mL of 4% sodium dodecyl sulfate (SDS), boiled for 20 min, and collected by centrifugation (12,000× *g*, 12 min). The precipitate was washed with 12 mL of 1 M NaCl, followed by centrifugation under the same conditions; this washing step was repeated until the pellet became colorless. The final colorless pellet was suspended in 1–2 mL of deionized water, centrifuged again, and dissolved in 2 mL of deionized water containing 0.02% sodium azide (NaN_3_). The purified peptidoglycan was stored at 4 °C.

Peptidoglycan concentration was measured using a SmartSpec Plus spectrophotometer (Bio-Rad, Hercules, CA, USA). An absorbance of 1.0 at 540 nm corresponded approximately to 1 mg/mL of peptidoglycan.

To evaluate the hydrolytic activity of recombinant endolysins, proteins were separated by SDS-PAGE in gels containing 0.1 mg/mL purified peptidoglycan as a substrate. Following electrophoresis, the gel was rinsed several times gently with deionized water and then incubated in a renaturation buffer consisting of 25 mM Tris-HCl (pH 7.2) supplemented with 1% Triton X-100. After 1 h of incubation at 37 °C, the gel was washed several times with deionized water and finally stained with a 1% methylene blue solution.

### 4.4. Antimicrobial Activity Assay of LysSte134_1

The ability of endolysin LysSte134_1 to kill target bacteria was assessed by counting colony-forming units (CFUs) after incubation with LysSte134_1. *S. aureus* strain CEMTC 1685 (vancomycin-resistant) or *S. epidermidis* strain CEMTC 2058 (methicillin-resistant)) was freshly cultured to the exponential phase (OD_600_ = 0.5) and harvested by centrifugation at 4000× *g* for 5 min. The harvested cells were washed and resuspended in R-buffer (50 mM Tris-HCl, pH 8.0); the cell suspension was diluted to a titer of 10^7^ CFU/mL. LysSte134_1 was then diluted in R-buffer alone, in R-buffer with 10% (*v*/*v*) 1,3-propanediol without Zn^2+^, in R-buffer with ZnCl_2_ at final concentrations of 1, 2.5, 5, or 10 µM, or in R-buffer with 10 µM ZnCl_2_ combined with 10% (*v*/*v*) 1,3-propanediol. Cells without LysSte134_1 were used as a control. A total of 100 µL of cell suspension was mixed with serial dilutions of LysSte134_1 in a 96-well plate and incubated at 37 °C. Aliquots of the suspensions were plated onto LB agar plates, and colonies were counted the following day.

### 4.5. Hemolytic Activity Assay

The hemolytic activity of LysSte134_1 was evaluated using human and mouse erythrocytes. Human erythrocytes were obtained from healthy donors. Murine erythrocytes were obtained from BALB/c mice. Freshly collected blood was diluted in appropriate anticoagulant and washed three times with phosphate-buffered saline (PBS) by centrifugation to obtain an 8% erythrocyte suspension. LysSte134_1 was diluted in PBS to prepare a series of two-fold serial dilutions starting from 100 µg/mL. Equal volumes of erythrocyte suspension and LysSte134_1 solutions were mixed and incubated at 37 °C for either 1 h or 6 h. After incubation, samples were centrifuged and the absorbance of the supernatants was measured at 540 nm to assess hemoglobin release. PBS alone and a lytic agent (1% Tween-20) were used as negative and positive controls, respectively. Hemolysis was expressed as a percentage of the positive control.

### 4.6. Effectivity of Endolysin LysSte134_1 in Staphylococcal Mice Wound Infection Model

Female BALB/c mice (6–8 weeks old, 16–18 g) were acclimated for 7 days and then randomly divided into six groups (*n* = 6 per group): intact uninfected animals without a wound (Intact); animals with a wound that was not infected (Non-infected wound); infected animals left untreated (Untreated); infected animals treated with vehicle (0.9% NaCl); infected animals treated with the endolysin-free composition (Composition); and infected animals treated with the LysSte134_1-containing composition (LysSte134_1). To minimize baseline differences, randomization was performed after body weight measurement, and group allocation was checked to ensure comparable mean body weight across groups at study start. To reduce the risk of bias, the investigators responsible for wound homogenization, CFU counting and hematological/cytokine measurements were blinded to group allocation. Samples were labeled with numeric codes that did not reveal the treatment group, and decoding was performed only after completion of data analysis. The mice were shaved, and 50 µL of sterile saline was injected intradermally to create necrotic wounds. Three days later, 50 µL of a MRSA strain CEMTC 675 suspension (5 × 10^7^ CFU/animals) or *S. epidermidis* strain CEMTC 2058 (5 × 10^7^ CFU/animals) was applied to each wound and allowed to dry naturally. In the Non-infected wound group, a wound was created in the same way but sterile saline was applied instead of the bacterial suspension. Intact animals received neither a wound nor any treatment and served as the baseline reference group. Seventy-two hours post-infection, infected animals were treated once daily for 5 days with 100 µL of the assigned formulation: the LysSte134_1-containing composition (1 mg/mL; LysSte134_1), the endolysin-free composition (Composition), 0.9% NaCl (vehicle; NaCl), or no treatment (Untreated). On day 14 post-infection, wound tissues were collected, homogenized in PBS, and used for CFU counting to assess bacterial load. Blood samples were collected on days 2 and 4 after the start of treatment. Serum cytokine concentrations were measured in three of the six experimental groups (Untreated, LysSte134_1-treated and Non-infected wound); *n* = 3 mice per group for the ELISA analysis. The samples were used for ELISA detection of cytokine levels (TNF-α, IL-6, IL-10 and IL-17) using commercial kits (Cloud Clone Inc., China), as well as for extended hematological analysis. Wound dimensions were measured at the beginning of treatment and on the final day of observation using a sterile disposable transparent ruler placed adjacent to the wound without contacting the wound bed. The longest axis of the wound was recorded as the length and the perpendicular axis as the width, both in millimeters. Wound area was approximated as length × width.

### 4.7. Statistical Analyses

Statistical analyses were performed using Statistica 10 software (StatSoft Inc., Tulsa, OK, USA). In vitro killing assays and hemolytic activity data were analyzed by one-way analysis of variance (ANOVA) followed by Tukey’s HSD post hoc test for pairwise comparisons. Bacterial burden in wounds was analyzed by two-way ANOVA followed by Tukey’s HSD post hoc test. Body weight time course and serum cytokine concentrations, obtained from the same animals at multiple time points, were analyzed by two-way repeated-measures ANOVA with between-subjects factor Group and within-subjects factor Time or Day; the Greenhouse–Geisser correction was applied to within-subjects terms when sphericity was violated (Mauchly’s test). Post hoc pairwise comparisons for the time-course data were performed by Holm-corrected pairwise tests, by Tukey’s HSD for between-group simple effects and by paired *t*-tests for within-group day 2 vs. day 4 changes. In vitro killing assays were performed in three biological replicates with three technical replicates each; in vivo endpoints were assessed in *n* = 6 animals per group, and serum cytokine measurements in *n* = 3 mice per group. This sample size was chosen on the basis of effect sizes (≥2 log reduction in wound CFU) reported in comparable murine endolysin studies, which are reliably detected with *n* = 5–6 per group at α = 0.05 and power = 0.80. Error bars in all figures represent the mean ± standard deviation (SD). Exact *p*-values are reported in the figure legends and in Table 3 legend for the in vivo analyses; for the in vitro killing assay (Figure 2), differences exceeding the detection threshold are reported as *p* < 0.0001.

## Figures and Tables

**Table 1:** Blood parameters on day 2 after treatment initiation.

Group	WBC × 10^9^/L	LYM%	NEU%	EOS%	RBC × 10^12^/L
**Untreated infected**	22.01 ± 17.80	55.33 ± 48.00	3.75 ± 3.25	6.14 ± 5.09	8.90 ± 4.51
**LysSte134_1-treated**	17.90 ± 6.20	80.67 ± 11.56	12.94 ± 13.01	3.66 ± 1.79	7.80 ± 1.85
**Non-infected wound**	22.19 ± 10.13	80.54 ± 4.20	11.81 ± 7.89	5.43 ± 3.34	11.60 ± 1.30
**Intact**	19.27 ± 11.38	49.53± 16.44	8.22 ± 4.13	1.37 ± 0.31	7.33 ± 1.41

**Table 2:** Blood parameters on day 4 after treatment initiation.

Group	WBC × 10^9^/L	LYM%	NEU%	EOS%	RBC × 10^12^/L
**Untreated infected**	6.92 ± 4.54	83.92 ± 8.82	9.08 ± 9.84	4.86 ± 2.37	7.34 ± 2.92
**LysSte134_1-treated**	15.07 ± 3.78	58.68 ± 46.31	23.35 ± 39.72	15.41 ± 12.27	4.22 ± 1.92
**Non-infected wound**	12.26 ± 7.62	75.64 ± 8.06	16.25 ± 12.04	5.19 ± 3.92	8.79 ± 2.43
**Intact**	17.17 ± 10.88	40.72± 11.22	7.72 ± 5.88	1.61± 0.11	8.87 ± 2.16

**Table 3:** Wound area at the beginning and end of treatment. Wound area (mm^2^) was measured on the first and last days of treatment in the NaCl, Untreated, Composition, LysSte134_1, and Non-infected wound groups (*n* = 6 per group). Intact animals had no wounds and were excluded from the analysis. Data are presented as mean ± SD. Wound area decreased significantly in the Untreated (*p* = 0.002), LysSte134_1 (*p* = 0.004), and Non-infected wound (*p* < 0.001) groups, but not in the NaCl (*p* = 0.157) or Composition (*p* = 0.363) groups. The greatest reductions were observed in the Non-infected wound (43.8%) and LysSte134_1 (34.1%) groups. Percentage wound-area reduction differed significantly among groups (one-way ANOVA, F(4,25) = 12.62F(4,25) = 12.62F(4,25) = 12.62, *p* < 0.0001). The LysSte134_1 group showed greater wound-area reduction than the Composition (*p* = 0.008), NaCl (*p* = 0.011), and Untreated (*p* = 0.012) groups, but did not differ from the Non-infected wound group (*p* = 0.485).

Group	Wound Area on the First Day of Treatment, mm^2^, mm^2^	Wound Area on the Last Day of Observation, mm^2^
***S. aureus***		
**LysSte134_1**	84.17 ± 9.74	62.91 ± 6.74
**NaCl**	89.31 ± 16.99	82.90 ± 17.11
**Composition**	89.33 ± 13.04	82.54 ± 15.26
**Untreated**	85.43 ± 8.85	75.48 ± 6.98
**Non-infected wound**	75.21 ± 9.12	42.80 ± 10.16
***S. epidermidis***		
**LysSte134_1**	90.4 ± 27.62	56.8 ± 16.30
**NaCl**	80.17 ± 21.78	69.33 ± 9.20
**Composition**	86.00 ± 20.86	80.67 ± 25.13
**Untreated**	95.33 ± 10.86	84.67 ± 8.91
**Non-infected wound**	75.21 ± 9.12	42.80 ± 10.16

## Data Availability

Data are contained within the article and Supplementary Materials.

## Supplementary Materials

The following supporting information can be downloaded at: https://www.mdpi.com/article/10.3390/ijms27187989/s1.

## Abbreviations

**Abbreviation**	**Definition**
ANOVA	Analysis of variance
CEMTC	Collection of Extremophile Microorganisms and Type Cultures
CFU	Colony-forming units
CXCL-1	C-X-C motif chemokine ligand 1
ELISA	Enzyme-linked immunosorbent assay
EOS	Eosinophils
ESKAPE	*Enterococcus faecium*, *Staphylococcus aureus*, *Klebsiella pneumoniae*, *Acinetobacter baumannii*, *Pseudomonas aeruginosa*, *Enterobacter* spp.
ESKAPEE	ESKAPE pathogens plus *Escherichia coli*
EUCAST	European Committee on Antimicrobial Susceptibility Testing
HEPES	4-(2-hydroxyethyl)-1-piperazineethanesulfonic acid
ICBFM SB RAS	Institute of Chemical Biology and Fundamental Medicine, Siberian Branch of the Russian Academy of Sciences
IL	Interleukin
IPTG	Isopropyl β-D-1-thiogalactopyranoside
LB	Luria–Bertani (lysogeny broth)
LYM	Lymphocytes
MDR	Multidrug-resistant
MRSA	Methicillin-resistant *Staphylococcus aureus*
MSCRAMM	Microbial surface components recognizing adhesive matrix molecules
NEU	Neutrophils
Ni-NTA	Nickel-nitrilotriacetic acid
OD_600_	Optical density at 600 nm
PAGE	Polyacrylamide gel electrophoresis
PBS	Phosphate-buffered saline
PIA	Polysaccharide intercellular adhesin
PNAG	Poly-*N*-acetylglucosamine
RBC	Red blood cells
16S rRNA	16S ribosomal RNA
SDS	Sodium dodecyl sulfate
SDS-PAGE	Sodium dodecyl sulfate polyacrylamide gel electrophoresis
TNF-α	Tumor necrosis factor alpha
VRSA	Vancomycin-resistant *Staphylococcus aureus*
WBC	White blood cells
WHO	World Health Organization

## References

[1] Touaitia R., Mairi A., Ibrahim N.A., Basher N.S., Idres T., Touati A. (2025). Staphylococcus Aureus: A Review of the Pathogenesis and Virulence Mechanisms. Antibiotics.

[2] Au S., Cruz W.D., Lala M., Karthikeyan S., Venketaraman V. (2026). The Evolution of Symbiosis in Staphylococcus Epidermidis: From a Protective Mutualist to a Parasitic Pathogen. Biomolecules.

[3] Patil A., Banerji R., Kanojiya P., Saroj S.D. (2021). Foodborne ESKAPE Biofilms and Antimicrobial Resistance: Lessons Learned from Clinical Isolates. Pathog. Glob. Health.

[4] Martínez-Santos V.I., Torres-Añorve D.A., Echániz-Aviles G., Parra-Rojas I., Ramírez-Peralta A., Castro-Alarcón N. (2022). Characterization of Staphylococcus Epidermidis Clinical Isolates from Hospitalized Patients with Bloodstream Infection Obtained in Two Time Periods. PeerJ.

[5] Bereanu A.-S., Bereanu R., Mohor C., Vintilă B.I., Codru I.R., Olteanu C., Sava M. (2024). Prevalence of Infections and Antimicrobial Resistance of ESKAPE Group Bacteria Isolated from Patients Admitted to the Intensive Care Unit of a County Emergency Hospital in Romania. Antibiotics.

[6] Ahmadunissah A., Aazmi S., Hamid U.M.A., Abdul-Aziz A. (2022). Multidrug Resistance of Staphylococcus Epidermidis: An Emerging Threat to Global Health. J. App. Pharm. Sci..

[7] Eriksson E., Liu P.Y., Schultz G.S., Martins-Green M.M., Tanaka R., Weir D., Gould L.J., Armstrong D.G., Gibbons G.W., Wolcott R. (2022). Chronic Wounds: Treatment Consensus. Wound Repair Regen..

[8] Redmond M.C., Gethin G., Finn D.P. (2025). A Review of Chronic Wounds and Their Impact on Negative Affect, Cognition, and Quality of Life. Int. Wound J..

[9] Falcone M., De Angelis B., Pea F., Scalise A., Stefani S., Tasinato R., Zanetti O., Dalla Paola L. (2021). Challenges in the Management of Chronic Wound Infections. J. Glob. Antimicrob. Resist..

[10] Versey Z., da Cruz Nizer W.S., Russell E., Zigic S., DeZeeuw K.G., Marek J.E., Overhage J., Cassol E. (2021). Biofilm-Innate Immune Interface: Contribution to Chronic Wound Formation. Front. Immunol..

[11] Molasy B., Wrzosek M. (2026). The Wound Microbiome in Chronic Wounds: A Biomarker and Therapeutic Target. J. Appl. Microbiol..

[12] Jiang S., Matuszewska M., Chen M., Hong Y., Chen Y., Wang Z., Zhuang H., Sun L., Zhu F., Wang H. (2025). Emergence and Spread of ST5 Methicillin-Resistant *Staphylococcus Aureus* with Accessory Gene Regulator Dysfunction: Genomic Insights and Antibiotic Resistance. Microbiol. Res..

[13] Chaffin D.O., Taylor D., Skerrett S.J., Rubens C.E. (2012). Changes in the Staphylococcus Aureus Transcriptome during Early Adaptation to the Lung. PLoS ONE.

[14] Raziyeva K., Kim Y., Zharkinbekov Z., Kassymbek K., Jimi S., Saparov A. (2021). Immunology of Acute and Chronic Wound Healing. Biomolecules.

[15] Al-Taweel R., Hammad A.S., Tajammul A., Crovella S., Al-Asmakh M. (2025). Wounds and the Microbiota: The Healing Interplay Between Host and Microbial Communities. Int. J. Mol. Sci..

[16] Valour F., Trouillet-Assant S., Rasigade J.-P., Lustig S., Chanard E., Meugnier H., Tigaud S., Vandenesch F., Etienne J., Ferry T. (2013). Staphylococcus Epidermidis in Orthopedic Device Infections: The Role of Bacterial Internalization in Human Osteoblasts and Biofilm Formation. PLoS ONE.

[17] Sabaté Brescó M., Harris L.G., Thompson K., Stanic B., Morgenstern M., O’Mahony L., Richards R.G., Moriarty T.F. (2017). Pathogenic Mechanisms and Host Interactions in Staphylococcus Epidermidis Device-Related Infection. Front. Microbiol..

[18] Månsson E., Tevell S., Nilsdotter-Augustinsson Å., Johannesen T.B., Sundqvist M., Stegger M., Söderquist B. (2021). Methicillin-Resistant Staphylococcus Epidermidis Lineages in the Nasal and Skin Microbiota of Patients Planned for Arthroplasty Surgery. Microorganisms.

[19] Arciola C.R., Campoccia D., Ravaioli S., Montanaro L. (2015). Polysaccharide Intercellular Adhesin in Biofilm: Structural and Regulatory Aspects. Front. Cell. Infect. Microbiol..

[20] Ahmad S., Rahman H., Qasim M., Nawab J., Alzahrani K.J., Alsharif K.F., Alzahrani F.M. (2022). Staphylococcus Epidermidis Pathogenesis: Interplay of icaADBC Operon and MSCRAMMs in Biofilm Formation of Isolates from Pediatric Bacteremia in Peshawar, Pakistan. Medicina.

[21] Williamson D.A., Carter G.P., Howden B.P. (2017). Current and Emerging Topical Antibacterials and Antiseptics: Agents, Action, and Resistance Patterns. Clin. Microbiol. Rev..

[22] Razdan K., Garcia-Lara J., Sinha V.R., Singh K.K. (2022). Pharmaceutical Strategies for the Treatment of Bacterial Biofilms in Chronic Wounds. Drug Discov. Today.

[23] Cavallo I., Sivori F., Mastrofrancesco A., Abril E., Pontone M., Di Domenico E.G., Pimpinelli F. (2024). Bacterial Biofilm in Chronic Wounds and Possible Therapeutic Approaches. Biology.

[24] Hurlow J., Wolcott R.D., Bowler P.G. (2025). Clinical Management of Chronic Wound Infections: The Battle against Biofilm. Wound Repair Regen..

[25] Qian H., Zheng Y. (2026). Phage Endolysins as Alternative Antimicrobials: Mechanisms, Clinical Progress, and Emerging Resistance Frameworks. Front. Microbiol..

[26] Abdelrahman F., Easwaran M., Daramola O.I., Ragab S., Lynch S., Oduselu T.J., Khan F.M., Ayobami A., Adnan F., Torrents E. (2021). Phage-Encoded Endolysins. Antibiotics.

[27] Lindsay S., Oates A., Bourdillon K. (2017). The Detrimental Impact of Extracellular Bacterial Proteases on Wound Healing. Int. Wound J..

[28] Iwasaki T., Uchiyama R., Nosaka K. (2023). Difference in Anti-Microbial Activity of Propan-1,3-Diol and Propylene Glycol. Chem. Pharm. Bull..

[29] Li B., Gao W., Pan Y., Yao Y., Liu G. (2024). Progress in 1,3-Propanediol Biosynthesis. Front. Bioeng. Biotechnol..

[30] Golosova N.N., Matveev A.L., Tikunova N.V., Khlusevich Y.A., Kozlova Y.N., Morozova V.V., Babkin I.V., Ushakova T.A., Zhirakovskaya E.V., Panina E.A. (2024). Bacteriophage vB_SepP_134 and Endolysin LysSte_134_1 as Potential Staphylococcus-Biofilm-Removing Biological Agents. Viruses.

[31] Dhar Y., Han Y. (2020). Current Developments in Biofilm Treatments: Wound and Implant Infections. Eng. Regen..

[32] Taati Moghadam M., Khoshbayan A., Chegini Z., Farahani I., Shariati A. (2020). Bacteriophages, a New Therapeutic Solution for Inhibiting Multidrug-Resistant Bacteria Causing Wound Infection: Lesson from Animal Models and Clinical Trials. Drug Des. Dev. Ther..

[33] Wu N., Zhu T. (2021). Potential of Therapeutic Bacteriophages in Nosocomial Infection Management. Front. Microbiol..

[34] Liu B., Guo Q., Li Z., Guo X., Liu X. (2023). Bacteriophage Endolysin: A Powerful Weapon to Control Bacterial Biofilms. Protein J..

[35] Azeredo J., García P., Drulis-Kawa Z. (2021). Targeting Biofilms Using Phages and Their Enzymes. Curr. Opin. Biotechnol..

[36] Rogovski P., Cadamuro R.D., da Silva R., de Souza E.B., Bonatto C., Viancelli A., Michelon W., Elmahdy E.M., Treichel H., Rodríguez-Lázaro D. (2021). Uses of Bacteriophages as Bacterial Control Tools and Environmental Safety Indicators. Front. Microbiol..

[37] Shield C.G., Swift B.M.C., McHugh T.D., Dedrick R.M., Hatfull G.F., Satta G. (2021). Application of Bacteriophages for Mycobacterial Infections, from Diagnosis to Treatment. Microorganisms.

[38] Ioannou P., Baliou S., Samonis G. (2023). Bacteriophages in Infectious Diseases and Beyond—A Narrative Review. Antibiotics.

[39] Garcia Torres S., Henrich D., Verboket R.D., Marzi I., Hahne G., Kempf V.A.J., Göttig S. (2025). Bactericidal Effect of a Novel Phage Endolysin Targeting Multi-Drug-Resistant Acinetobacter Baumannii. Antibiotics.

[40] Zinsli L.V., Sobieraj A.M., Du J., Ernst P., Meile S., Kilcher S., Iseli C., Keller A.P., Dreier B., Mittl P.R.E. (2025). Heterodimerization of Staphylococcal Phage φ2638A Endolysin Isoforms and Their Functional Role in Bacterial Lysis. microLife.

[41] Wen Q., Huang X., Ma W., Chen Y., Wang L., Ma Y., Chen X. (2025). Characterization of a Phage Endolysin LysLFP01 and Its Antibacterial Activity. Int. J. Food Microbiol..

[42] Prajapati N., Prajapati D., Patani A., Tashbaev S., Ugli G.G.S., Patel A. (2025). Bacteriophage Endolysins. Enzymes.

[43] Zhang Y., Deng C., Liu Y., Lan W., Zhao Y., Sun X. (2026). A New Endolysin Lys59: A Broad-Spectrum Phage Endolysin Targeting Both Gram-Negative and Gram-Positive Bacteria. Microorganisms.

[44] Zhang Y., Lan W., Sun X. (2025). Phage-Encoded Depolymerases and Endolysins as Prospective Strategies to Combat Multidrug-Resistant Klebsiella Pneumoniae. Int. J. Biol. Macromol..

[45] Shahrour H., Alves Ferreira D., Fitzgerald-Hughes D., O’Gara J.P., Coffey A., O’Neill E. (2025). Use of the Bacteriophage-Derived Endolysin CHAPK-SH3blys as a Potent Novel Treatment for Biofilm-Associated Staphylococcus Aureus Wound Infections. Microbiol. Spectr..

[46] Liu H., Wei X., Peng H., Yang Y., Hu Z., Rao Y., Wang Z., Dou J., Huang X., Hu Q. (2024). LysSYL-Loaded pH-Switchable Self-Assembling Peptide Hydrogels Promote Methicillin-Resistant Staphylococcus Aureus Elimination and Wound Healing. Adv. Mater..

[47] Abdulkadieva M., Slonova D., Litvinenko V., Antonova N., Mazunina E., Sobyanin K., Guseva T., Parshina O., Domnin P., Poloskov V. (2025). Wound-Healing Potential of Engineered Lysin GRC-ML07 in Pseudomonas Aeruginosa Infected Wounds in Immunocompromised Mice. Antibiotics.

[48] Euler C.W., Raz A., Hernandez A., Serrano A., Xu S., Andersson M., Zou G., Zhang Y., Fischetti V.A., Li J. (2023). PlyKp104, a Novel Phage Lysin for the Treatment of Klebsiella Pneumoniae, Pseudomonas Aeruginosa, and Other Gram-Negative ESKAPE Pathogens. Antimicrob. Agents Chemother..

[49] Vasina D.V., Antonova N.P., Gushchin V.A., Aleshkin A.V., Fursov M.V., Fursova A.D., Gancheva P.G., Grigoriev I.V., Grinkevich P., Kondratev A.V. (2024). Development of Novel Antimicrobials with Engineered Endolysin LysECD7-SMAP to Combat Gram-Negative Bacterial Infections. J. Biomed. Sci..

[50] Eichenseher F., Herpers B.L., Badoux P., Leyva-Castillo J.M., Geha R.S., van der Zwart M., McKellar J., Janssen F., de Rooij B., Selvakumar L. (2022). Linker-Improved Chimeric Endolysin Selectively Kills Staphylococcus Aureus In Vitro, on Reconstituted Human Epidermis, and in a Murine Model of Skin Infection. Antimicrob. Agents Chemother..

[51] Haddad Kashani H., Schmelcher M., Sabzalipoor H., Seyed Hosseini E., Moniri R. (2018). Recombinant Endolysins as Potential Therapeutics against Antibiotic-Resistant Staphylococcus aureus: Current Status of Research and Novel Delivery Strategies. Clin. Microbiol. Rev..

[52] Wilkinson H.N., Stafford A.R., Rudden M., Rocha N.D.C., Kidd A.S., Iveson S., Bell A.L., Hart J., Duarte A., Frieling J. (2024). Selective Depletion of Staphylococcus aureus Restores the Skin Microbiome and Accelerates Tissue Repair after Injury. J. Investig. Dermatol..

