# Supplementary material for: Endolysin LysSte134_1 Reduces Staphylococcus Bacterial Load in Infected Wounds In Vivo
Source: Int J Mol Sci. 2026 Sep 8;27(18):7989. doi: 10.3390/ijms27187989 (PMC13606704; doi:10.3390/ijms27187989)
Supplement: Supplementary file 1 [file ijms-27-07989-s001.zip › ijms-4465610-supplementary.pdf]

Table S1. Characteristics of *Staphylococcus* strains.

CEMTC	Sample source	β-lactams	Aminoglycosides	Macrolides	Lincosamides	Fluoroquinolones	Chloramphenicol	Glycopeptides	Other antibiotics	Antibiotic resistance
S. aureus										
**675**	bedsore	FOX	CN	E	DA					MDR
**1685**	throat swab							VA		R
S. epidermidis										
**301**	armpit skin									S
**2058**	fluid from the joint	P								R
**3441**	skin scraping									S
S. auricularis										
**2738**	ear swab, otitis				DA					R
S.capitis										
**1590**	urine, chronic infection									S
**2904**	scraping from the auricle									S
S. caprae										
**1849**	scraping from the auricle									S
**2305**	pure culture									S
S. simulans										
**1728**	diabetic foot ulcer									S
S.hominis										
**1702**	nasal swab									S
S. warneri										
**1997**	dried deer stomach									S
**3911**	throat swab									S
S. felis										
**2998**	eye smear									S

Abbreviations: MDR – Multi Drug Resistance; R – Resistance; S – Sensitive; AK – Amikacin; CN – Gentamicin; DA – Clindamycin; FOX – Cefoxitin; E – Erytromycin; P – Penicillin; VA – Vancomycin.
